# Mirabegron administration for the prevention of ureteral injuries during ureteral access sheath insertion

**DOI:** 10.1007/s00345-026-06225-3

**Published:** 2026-01-27

**Authors:** Osman Ermiş, Kubilay Sabuncu, Çağrı Kaçtan, Burak Karakuş, Bahattin Sürmeli, Mustafa Yücel Boz, Rahim Horuz

**Affiliations:** Department of Urology, Medipol Mega University Hospital, Istanbul, Turkey

**Keywords:** Kidney stone, Mirabegron, Retrograde intrarenal surgery, Ureteral access sheath, Ureter injuries

## Abstract

**Purpose:**

Urolithiasis is a common condition in urological practice. Retrograde intrarenal surgery (RIRS) is widely accepted as a safe and effective treatment modality. However, acute ureteral injuries during ureteral access sheath (UAS) placement remain a significant concern. Although beta-adrenergic receptors have been identified in the ureteral wall, studies investigating beta-agonists for ureteral protection are lacking. This study aimed to assess whether short-term preoperative administration of mirabegron, a beta-3 adrenoreceptor agonist, can reduce the incidence of UAS-related ureteral injuries during RIRS.

**Methods:**

In this prospective non-randomized study, 60 patients undergoing RIRS were enrolled. Allocation was based on clinical indications due to ethical considerations: 30 patients with existing overactive bladder symptoms received preoperative mirabegron, while 30 patients served as controls. Baseline characteristics were comparable between the groups. Ureteral injuries were assessed endoscopically and graded using the Post-Ureteroscopic Lesion Scale (PULS).

**Results:**

The mirabegron group showed a lower incidence of high-grade ureteral injuries compared to the control group, but the difference was not statistically significant (*p* = 0.123). No adverse events related to mirabegron were reported. Limitations include the lack of randomization, modest sample size, and single-center design.

**Conclusions:**

Short-term preoperative mirabegron use appears to be safe. While our results suggest a potential trend towards reducing high-grade ureteral injuries, this did not reach statistical significance, likely due to the limited sample size of this preliminary cohort. Further large-scale, multicenter studies with longer follow-up are necessary to confirm these findings.

## Introduction

Urinary stone disease has long been recognized as a major global health concern, attracting considerable attention and inspiring extensive research into diagnosis and treatment strategies. Epidemiological studies have demonstrated that the prevalence of urinary stones varies across geographical regions, ranging from 1% to 20% [1]. In Turkey, which lies within the so-called “stone belt,” the estimated prevalence is approximately 15% [2]. Clinically, urinary stone disease presents with a wide spectrum of manifestations, from asymptomatic cases to severe obstructive events that can lead to life-threatening septic complications.

Retrograde intrarenal surgery (RIRS) has emerged as a well-established and minimally invasive treatment option for urinary calculi [3]. Recent technological advancements have significantly enhanced the efficacy and popularity of RIRS. However, procedural complications and variations in technique continue to limit its widespread adoption. High-quality, evidence-based clinical data are essential to validate the safety and effectiveness of RIRS for broader application [4]. One of the primary concerns during RIRS is the risk of acute ureteral wall injuries associated with the insertion of the ureteral access sheath (UAS). The UAS facilitates rapid and repeated access to the upper urinary tract, enabling improved irrigation, maintaining low intrarenal pressures, and enhancing visualization during surgery.

Despite these advantages, UAS placement carries a substantial risk of ureteral injury in any grade according to ureteral wall injury grading systems, with reported incidences ranging between 30.4% and 46.5% [5, 6]. Several preventive strategies have been proposed, including preoperative placement of double-J (DJ) stents, short-term administration of alpha-adrenergic blockers or phosphodiesterase type 5 (PDE5) inhibitors, and intraoperative balloon dilation [7–9]. These methods primarily aim to improve ureteral compliance or lower intraureteral pressure to facilitate safer UAS advancement. Additionally, histological studies have identified the presence of beta-adrenergic receptors (β-ARs) alongside alpha-adrenergic receptors (α-ARs) within the human ureter [10, 11].

While pharmacological ureteral relaxation via alpha-blockade has been explored, there remains a paucity of research examining the potential benefits of beta-adrenergic receptor agonists for the prevention of UAS-related ureteral injuries. In this context, we conducted a prospective study to evaluate the effect of short-term preoperative administration of mirabegron, a selective beta-3 adrenergic receptor (β3-AR) agonist, on the incidence of ureteral injuries during UAS placement.

## Materials and methods

### Study design and ethics approval

This non-randomized prospective study was approved by the Non-Interventional Clinical Research Ethics Committee of Istanbul Medipol University (Decision No. 878, dated 14/10/2022). Written informed consent was obtained from all participants prior to enrollment.

### Patient selection

The sample size calculation for the current study was based on the incidence of ureteral wall injury associated with ureteral access sheath (UAS) insertion reported by Traxer and Thomas, in which an overall injury rate of 46.5% was observed. Assuming a 30% absolute difference in the rate of ureteral wall injury between groups, with a two-sided α value of 0.05 and a desired statistical power of 0.8, the required sample size was estimated to be 45 patients per group (90 in total) [6].

Due to the practical constraints of patient recruitment, 30 patients were included in each group, resulting in a total of 60 patients. A post hoc power analysis, using the same assumptions for expected effect size and alpha level, indicated that the achieved statistical power was approximately 73%. The achieved statistical power was approximately 73%, which is slightly lower than the initially targeted 80%. While this may modestly increase the likelihood of a type II error, the findings of this study still provide valuable insights and should be interpreted in the context of this limitation.

A total of 60 patients were included, divided equally into a mirabegron group and a control group (30 patients each). Eligible patients were adults (> 18 years) requiring RIRS with UAS placement for treatment of upper ureteral or renal stones. Exclusion criteria included prior use of alpha-blockers, presence of ureteral stones at other locations, known ureteral strictures, history of ipsilateral ureteral surgery, anatomical anomalies (e.g., horseshoe kidney, duplicated ureter), and known or suspected allergy to mirabegron. Patients with positive preoperative urine cultures were also excluded. Patients who were not able to be inserted due to any reason UAS were excluded.

Randomization was not applied due to ethical considerations. Allocations were based on clinical indications: patients with irritative lower urinary tract symptoms consistent with overactive bladder required medical management and were therefore selected for the mirabegron group and received mirabegron 50 mg orally once daily for 7 days preoperatively, achieving steady-state plasma concentrations as supported by prior studies [12, 13]. Patients who have uncontrolled hypertension and/or arrhythmias were not selected for the mirabegron group. Patients experiencing intolerable pain or acute obstructive symptoms before the planned surgery were excluded from the study and treated earlier if necessary. Adherence to medication and any side effects were monitored via emergency contact communication.

### Surgical technique and ureteral injury assessment

All surgeries were performed at a single center (Medipol Mega University Hospital) by an experienced surgeon under general anesthesia in the lithotomy position. A safety guidewire was routinely placed, followed by fluoroscopically guided insertion of a 9.5 F/11.5 F Cook^®^ UAS. In cases of excessive resistance, the procedure was aborted, a DJ stent was placed, and the patient was excluded from the study. RIRS was then completed. UAS removal and intraoperative ureteral injury assessment were performed under direct ureteroscopic visualization. All steps were recorded on video, reviewed by a second independent endourologist who was blinded to the patient group allocation. Ureteral injuries were graded according to the Post-Ureteroscopic Lesion Scale (PULS). In cases of discrepancy between the primary and secondary assessments, the final grade was determined by consensus.Postoperatively, a DJ stent was placed in all patients with injuries of grade 1 or higher. DJ stents were removed after 10 days in cases of minor injuries and after approximately 14 days in those with grade 2 or higher injuries.

Ureteral injuries were classified according to a five-grade system previously described [6]:

Grade 0: No injury or only mucosal petechiae.

Grade 1: Mucosal erosion or flap without muscular injury.

Grade 2: Injury involving mucosa and muscularis, with intact adventitia.

Grade 3: Full-thickness perforation involving adventitia.

Grade 4: Complete ureteral avulsion with total loss of continuity.

Grades 0–1 were defined as low-grade injuries, and grades 2–4 were defined as high-grade injuries. These groups were compared statistically.

### Secondary outcomes and statistical analysis

Secondary outcomes included demographic characteristics, adverse events related to mirabegron, surgical complications, hospital stay duration, stone-free rates, and postoperative pain scores assessed by a visual analog scale (VAS). Pain was evaluated three hours after surgery on a 0–10 scale.

Surgical complications were graded using the modified Clavien–Dindo classification system [14]. Stone-free status was confirmed intraoperatively through fluoroscopy and endoscopic inspection and verified by ultrasonography at one month postoperatively.

Statistical analysis was conducted using JAMOVI version 2.3.21 software. Categorical variables were presented as frequencies and percentages, while continuous variables were summarized as means ± standard deviation. Distribution normality was assessed using visual (histograms, plots) and analytical (Kolmogorov–Smirnov and Shapiro–Wilk) tests. The chi-square test was used for categorical comparisons. For continuous data, the Student’s t-test was used when normality assumptions were met; otherwise, the Mann–Whitney U test was applied. A p-value < 0.05 was considered statistically significant.

Language and style editing assistance was provided by ChatGPT (OpenAI, San Francisco, CA, USA) to improve grammar, readability, and consistency.

## Results

A total of 60 patients were included in the study. The mean age was 42.50 ± 11.20 years in the mirabegron group and 44.27 ± 13.69 years in the control group, with no statistically significant difference (*p* = 0.586). The mirabegron group consisted of 14 males and 16 females, while the control group included 19 males and 11 females; the gender distribution was not significantly different between groups (*p* = 0.194). No drug-related adverse events were observed in any patient receiving mirabegron.

The mean body mass index (BMI) was 25.77 ± 3.42 kg/m² in the mirabegron group and 26.56 ± 4.68 kg/m² in the control group (*p* = 0.457). Comorbidities were present in 10 patients (33%) in the mirabegron group and 11 patients (37%) in the control group (*p* = 0.787). The mean glomerular filtration rate (GFR) was 98.60 ± 19.32 ml/min/1.73 m² in the mirabegron group and 92.90 ± 22.34 ml/min/1.73 m² in the control group (*p* = 0.219). There were no significant differences between groups in stone size, stone location, or stone density measured by Hounsfield Unit (*p* > 0.05 for all) (Table 1).

**Table 1:**
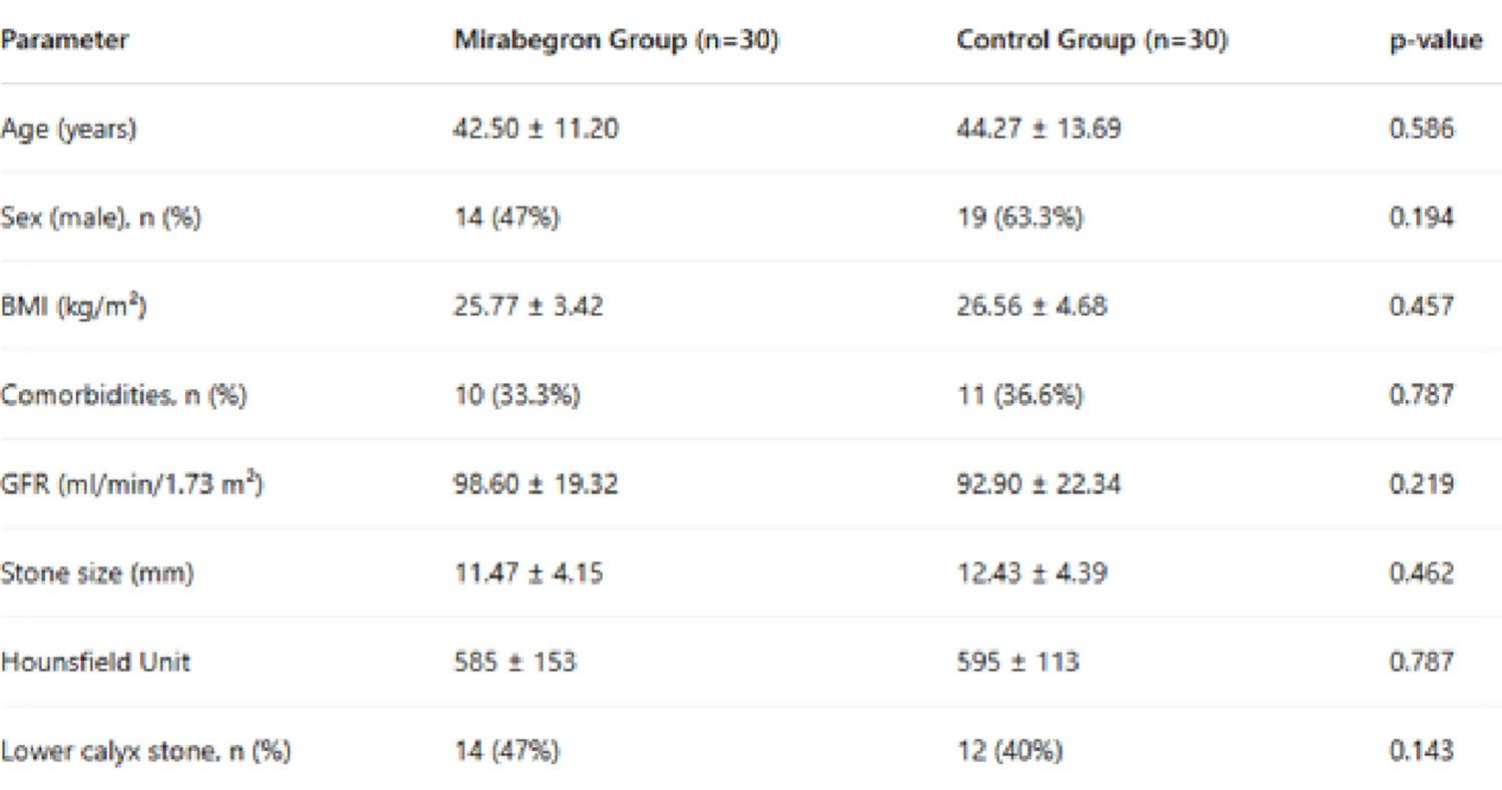
Demographic and preoperative characteristics of the participants

Ureteral injury of any grade was observed in 31 of the 60 patients (51.6%). In the mirabegron group, grade 0, 1, 2, and 3 injuries occurred in 17 (56.6%), 9 (30%), 3 (10%), and 1 (3.3%) patients, respectively; no grade 4 injuries were observed. In the control group, 11 patients (36.6%) had grade 0 injury, 10 (33.3%) had grade 1, 6 (20%) had grade 2, 2 (6.7%) had grade 3, and 1 (3.3%) had grade 4 injury (Table 2). Overall, high-grade ureteral injuries (grades 2–4) occurred in 13 patients (21.6%).

**Table 2:**
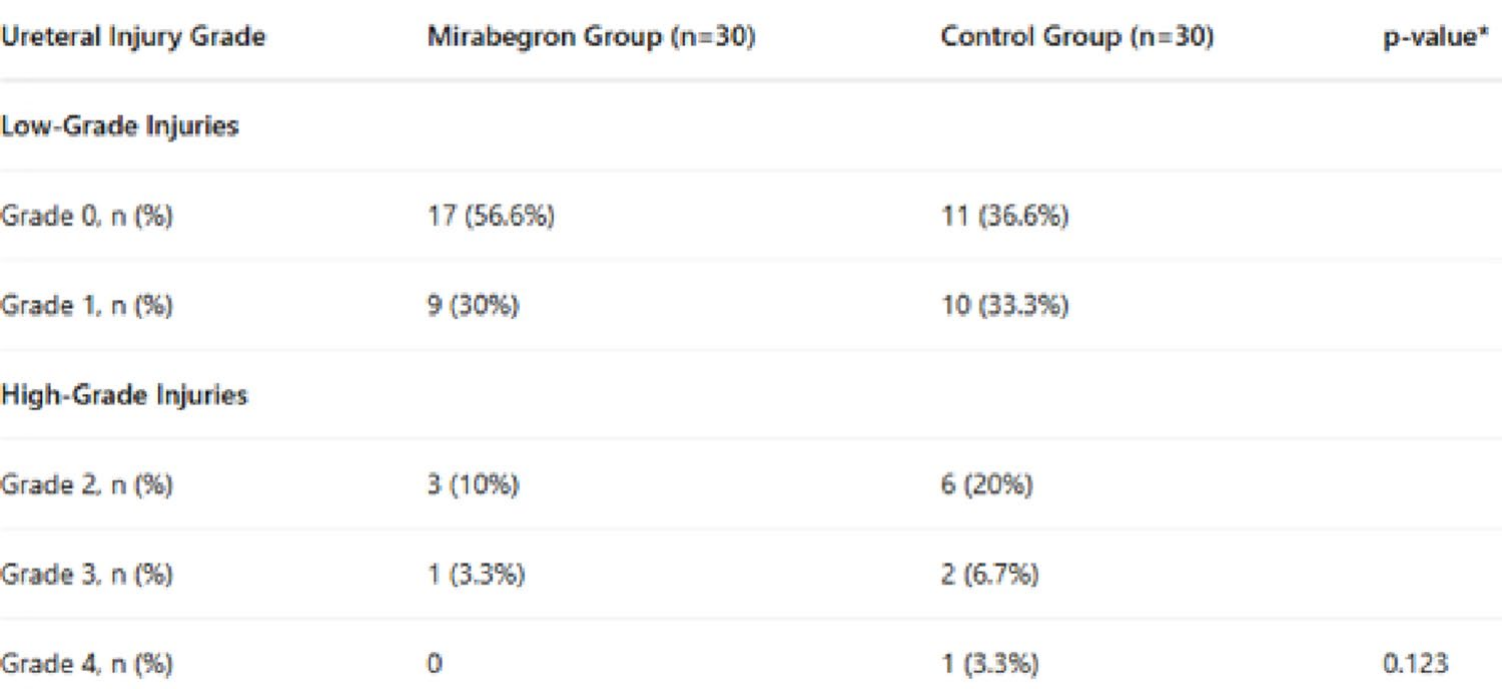
Ureteral injury scores by group

**Fig. 1:**
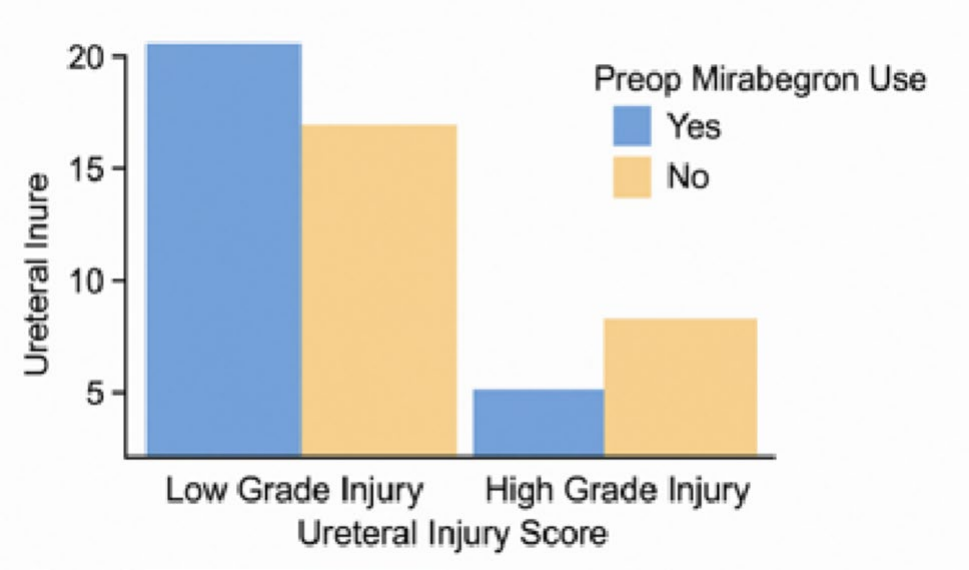
Preoperative mirabegron use and uretereal injury

While high-grade ureteral injury appeared less frequently in the mirabegron group, the difference was not statistically significant (Fig.1, *p* = 0.123).

Independent of mirabegron use, the relationship between sex and high-grade ureteral injury was analyzed. High-grade injuries were observed in 8 of 33 male patients (24.2%) and in 5 of 27 female patients (18.5%).

Postoperative pain was assessed using the visual analog scale (VAS) at 3 h postoperatively. Pain scores of 0–4 were classified as mild, and scores of 5–10 as moderate to severe. Mild pain was reported by 16 patients (53%) in the mirabegron group and 14 patients (47%) in the control group, with no statistically significant difference (*p* = 0.615) (Table 3).

**Table 3:**
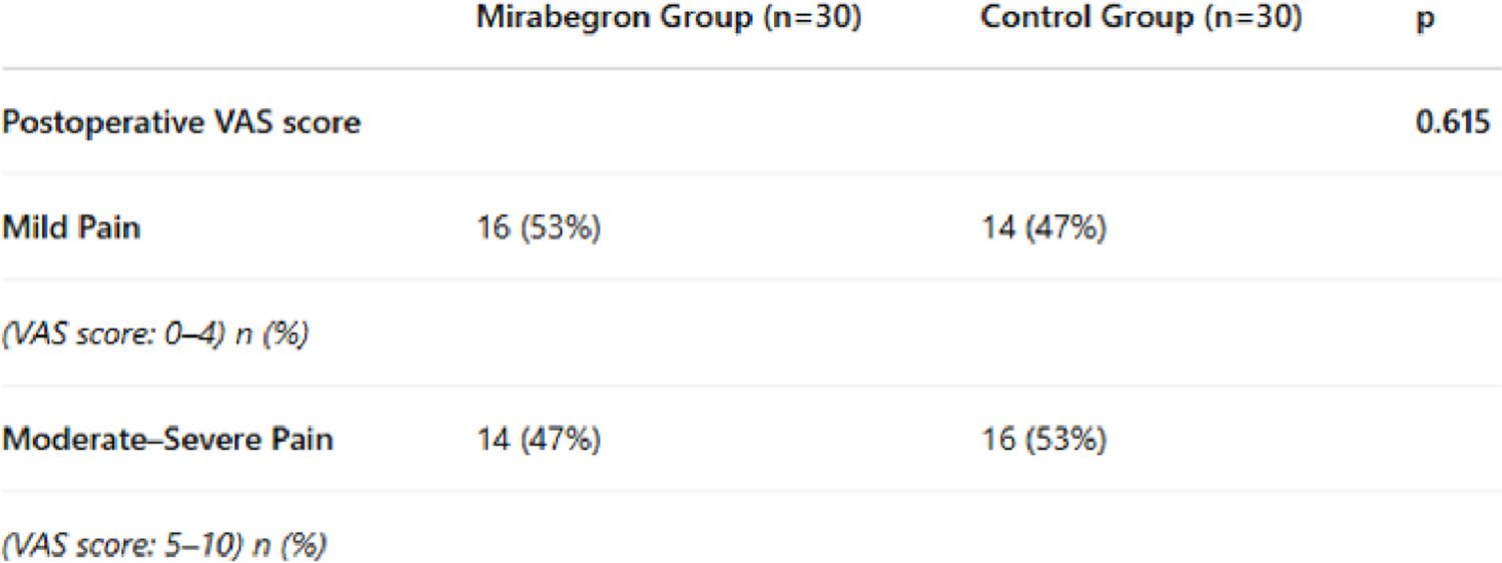
Postoperative VAS scores by group

The mean operative time was 83.50 ± 29.95 min in the mirabegron group and 91.37 ± 32.36 min in the control group (*p* = 0.386). The median postoperative hospital stay was 1 day (range: 1–1) in the mirabegron group and 1 day (range: 1–12) in the control group, with no statistically significant difference (*p* = 0.082). (Table 4).

**Table 4:**
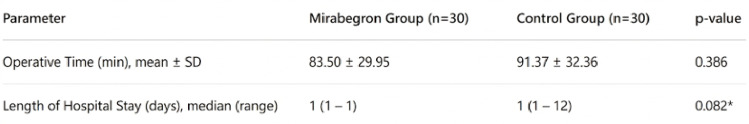
Operative and hospitalization durations by group

Postoperative complications were classified using the modified Clavien–Dindo system. In the mirabegron group, only grade 1 complications—those typically seen in standard postoperative recovery—were observed, with no additional interventions required. In the control group, 28 patients experienced grade 1 complications, while one patient experienced a grade 3b complication involving a distal ureteral avulsion, which required immediate open repair via ureteroneocystostomy. In this specific case, deviating from the standard protocol, the double-J stent was retained for 4 weeks and removed only after control CT urography confirmed watertight healing, and another patient experienced a grade 5 complication (death due to multiorgan failure following postoperative intensive care).

At the 1-month follow-up, stone-free status was assessed via ultrasonography in the 59 surviving patients. All patients in the mirabegron group (*n* = 30) were confirmed stone-free. In the control group (*n* = 29), one patient had a clinically significant residual stone fragment.

## Discussion

To our knowledge, this is the first prospective study to evaluate the use of the β3-adrenergic receptor agonist mirabegron for the prevention of ureteral wall injury during UAS insertion. Previous randomized controlled trials have primarily investigated α-adrenergic antagonists, while other studies have focused on the role of mirabegron in alleviating stent-related symptoms. None of these studies specifically addressed the preventive role of mirabegron in the context of UAS-related trauma.

UAS devices are generally 4 F to 8 F larger than the average adult ureteral diameter [15]. Excessive force during UAS insertion into a narrow ureter can result in mucosal injury, perforation, or even complete ureteral avulsion [16]. Furthermore, the intraluminal compression exerted by the sheath may compromise ureteral blood flow, leading to ischemia and the potential development of secondary strictures [17–19]. A previous prospective study involving 359 patients observed ureteral injuries of varying severity in nearly half of the cases [6]. Similarly, in our cohort, ureteral injury of any grade occurred in 51.6% of patients. Notably, the incidence of high-grade ureteral injury in our study (21.6%) was higher than previously reported (13.3%). This discrepancy is likely multifactorial. Firstly, our cohort had a 55% male predominance, and male gender is a known risk factor for ureteral resistance; indeed, our data showed a higher injury rate in men (24.2%) than women (18.5%). Secondly, the rigorous prospective nature of this study ensured that even borderline high-grade lesions were captured, whereas retrospective series may underreport such cases. We also observed one mortality (Clavien-Dindo Grade V) in the control group due to sepsis. This outcome is a critical reminder that despite being minimally invasive, RIRS with UAS placement involves real risks and demands close patient monitoring.

Several preventive strategies have been proposed to minimize UAS-related ureteral trauma, including passive dilation via preoperative double-J (DJ) stenting, semirigid ureteroscope-assisted dilation, balloon dilation of the distal ureter, and pharmacologic relaxation using alpha-adrenergic antagonists. Alpha-blockers have been shown to reduce intraureteral pressure and facilitate sheath insertion [8, 9, 20]. Importantly, in addition to alpha-adrenergic receptors, beta-adrenergic receptors (β-ARs) are also present within the ureter [10, 11]. Beta-agonists such as mirabegron have been demonstrated to induce ureteral wall relaxation and lower intraureteral pressure [11]. Based on this mechanism, mirabegron has been investigated for its ability to alleviate stent-related symptoms and enhance medical expulsive therapy for distal ureteral stones [21–25]. Currently, alpha-adrenergic antagonists (e.g., tamsulosin, silodosin) and PDE5 inhibitors (e.g., tadalafil) are the most investigated agents for facilitating UAS insertion. These drugs primarily work by inhibiting smooth muscle contraction. In contrast, mirabegron represents a different pharmacological approach by actively promoting relaxation via beta 3-adrenergic receptor stimulation and the cAMP signaling pathway. Although alpha-blockers remain the most widely used agents for this indication, the distinct mechanism of mirabegron suggests it could be a valuable alternative, particularly in patients who may not tolerate alpha-blockade. These pathophysiological principles formed the foundation for our hypothesis.

Although pre-stenting provides effective passive ureteral dilation and may reduce trauma [26], it is associated with adverse symptoms such as flank pain, urinary urgency, and dysuria [27], and it requires an additional procedure with associated costs. In contrast, short-term preoperative use of mirabegron offers a less invasive and potentially more cost-effective alternative. However, no study to date has directly compared the efficacy of pre-stenting, alpha-blockers, and mirabegron in preventing UAS-related injuries. Our results suggest a lower incidence of high-grade injuries in the mirabegron group, supporting the potential protective effect of β3-agonist therapy.

Previous studies have reported a higher incidence of high-grade ureteral injuries among male patients [6]. Although the underlying mechanism is not fully understood, possible explanations include increased psoas muscle tone or hormonal influences [6, 28]. In line with the existing literature, our findings also demonstrated a higher frequency of high-grade injuries among male patients.

Postoperative pain following RIRS is likely multifactorial, related to mucosal friction from the sheath, smooth muscle irritation, and fluctuating pressures within the pelvicalyceal system due to irrigation [29, 30]. It is plausible that narrower ureters subjected to UAS placement are associated with greater postoperative discomfort. Through activation of β-adrenergic receptors in the distal ureter, mirabegron may induce smooth muscle relaxation, lower intraureteral pressure, and facilitate UAS insertion, potentially reducing postoperative pain. Although this trend was observed in our study, the difference in VAS scores between groups did not reach statistical significance.

Our findings regarding operative time and hospital stay were consistent with those reported in the literature for similar patient populations undergoing RIRS. The complication rates and operative times in this study reflect the strict prospective recording of even minor (Clavien-Dindo Grade I) events and the statistical impact of outliers in a modest sample size. Major complications were rare, and prolonged operative times were limited to a few complex cases rather than reflecting routine surgical duration.

Worldwide, RIRS has become firmly established as a standard treatment for urinary stones, facilitated by growing surgical experience and technological improvements. Comparative studies have demonstrated that stone-free rates after RIRS are comparable to those achieved with extracorporeal shock wave lithotripsy (ESWL) and percutaneous nephrolithotomy (PCNL), with RIRS often requiring fewer retreatment sessions than ESWL [31–33]. In our study, among the 59 patients evaluated at follow-up, only one patient had a clinically significant residual fragment at the 1-month assessment, and no additional interventions were required in the remaining 58 patients.

Our study has several limitations. The primary limitation of this study is the non-randomized design, which introduces a significant selection bias. Since patients with existing OAB symptoms were preferentially allocated to the mirabegron group for ethical treatment purposes, the groups may not be perfectly comparable. Consequently, our results should be interpreted as exploratory and hypothesis-generating, rather than definitive evidence of efficacy. Crucially, the study achieved a post-hoc power of only 73% compared to the targeted 80%. This limitation implies that the lack of statistical significance (*p* = 0.123) regarding high-grade injuries may be attributable to the insufficient sample size rather than the absence of a true protective effect. Blinding was not performed due to the nature of the intervention, as both the surgical team and the patients were aware of preoperative mirabegron administration. All procedures were performed by a single experienced surgeon at a single center, which may limit generalizability to other settings with different levels of surgical expertise. Although a formal statistical test for interobserver agreement was not performed, we took specific measures to ensure consistency. All assessments were verified by a blinded independent endourologist, and any discrepancies were resolved through consensus. Moreover, the PULS system itself is a validated tool with proven reproducibility in the literature, which further supports the reliability of our data.Furthermore, the PULS system is a validated tool with established high inter-rater reliability in the literature, which supports the consistency of our findings.Furthermore, a 11.5 F UAS was used in all cases. Although sheath caliber may influence the risk of injury, studies have demonstrated comparable injury rates even with larger diameter sheaths [6, 8, 9, 20, 34].

A key limitation of our study is the short postoperative follow-up of one month. Current expert consensus states that six months is the minimum follow-up time to assess the presence of ureteral stricture (US) after endoscopic treatment, and prolonged follow-up is warranted for patient subgroups at higher risk of delayed-onset US [35].

A recent systematic review and meta-analysis encompassing numerous studies explicitly highlighted that the pooled rate of US significantly rises in studies with follow-up periods equal to or exceeding six months, confirming that US rates (3.1%) are much higher in long-term series compared to short-term series (0.4%) [35]. Consequently, an international Delphi consensus focused on iatrogenic ureteral strictures confirmed that a minimum follow-up duration of six months is mandatory for assessing US presence after endoscopic treatment [36].

The overall US rate following URS is reported between 0.3 and 4.9%, but specific risk factors greatly elevate this risk. The evidence indicates a strong association between US risk and intraoperative ureteral perforation (Odds Ratio [OR] = 7.1), the presence of impacted stones (OR = 7.47), and preoperative hydronephrosis (OR = 2.6). Moreover, US risk is associated with proximal ureteral stone location compared to distal stones (OR = 1.6). Given these high-risk factors and our limited follow-up, future large-scale studies with extended follow-up periods of at least six months are required to fully validate our findings regarding the potential protective role of mirabegron against US development.

## Conclusion

Ureteral wall injuries during UAS placement in RIRS remain a notable clinical concern. In this prospective study, preoperative administration of mirabegron appeared to be a safe and potentially effective method for reducing UAS-related ureteral trauma; however, the observed reduction did not reach statistical significance likely due to the limited sample size of the study. While passive dilation via preoperative double-J (DJ) stenting remains an effective strategy, it requires an additional procedure and incurs extra healthcare costs.

Although patients currently utilizing alpha-blockers for other purposes were excluded, mirabegron may represent a viable choice, necessitating future investigations for comparative analysis in this context. As a β3-adrenergic receptor agonist with established efficacy for overactive bladder, mirabegron may offer a novel preventive role in endourology.

Nevertheless, larger multicenter studies with greater statistical power and longer follow-up periods are needed to validate these preliminary findings and to clarify the potential role of mirabegron in the prevention of UAS-related ureteral injuries.

## Data Availability

The datasets generated and/or analysed during the current study are available from the corresponding author on reasonable request.
